# The influence of social and commercial pension insurance differences and social capital on the mental health of older adults—Microdata from China

**DOI:** 10.3389/fpubh.2022.1005257

**Published:** 2022-11-09

**Authors:** Kuang-Cheng Chai, Qiang Li, Chengsheng Jin, Yu-Jiao Lu, Zhenxin Cui, Xingxing He

**Affiliations:** ^1^Business School, Guilin University of Electronic Technology, Guilin, China; ^2^GUET Nanning Research Institute, Nanning, China

**Keywords:** Healthy China, pension insurance, social capital, mental health of the elderly, social pension insurance, commercial pension insurance

## Abstract

The number of older adults is rising rapidly in China. Various concerns such as chronic diseases, financial inadequacy, and a feeling of loneliness have adversely affected the mental health of older adults, and this has become an important public health and social issue. To realize healthy aging, the Nineteenth National People's Congress of China put forth the Healthy China strategy, speeding up the promotion activities of mental health and pension measures, carrying out public welfare pension insurance for the entire population, and contributing to the mental health of older adults. This study used data from China Family Panel Studies. This study mainly uses the random effect estimation method (random effect, RE) and the feasible generalized least squares estimation method (FGLS) to control for heterogeneity to explore the impact of social and commercial pension insurance on the mental health of older adults, the moderating effect of social capital on pension insurance, and the mental health of older adults. The results showed that social pension insurance is proportional to the mental health of older adults, whereas commercial pension insurance is inversely proportional to mental health. Social capital had a significant moderating effect on pension insurance. When a country develops an aging economy, the emphasis on social capital helps make targeted industrial development suggestions. The government's expansion of insurance coverage is crucial for improving the mental health of older adults.

## Introduction

With the advancement of economic development and urbanization, mental and psychological disorders have become key risk factors for non-communicable diseases and are the main components of the global disease burden (1–3). According to a survey, between 2013 and 2015, the weighted prevalence of any disorder (excluding dementia) during the 12 months and participants' entire lifetime before the interview was 9.3 and 16.6%, respectively. Anxiety disorders were the most common class of disorders, both during the 12 months before the interview (weighted prevalence 5.0%) and in participants' lifetimes (7.6%). The weighted prevalence of dementia in people aged 65 years or older is 5.6% (4).

Mental health has become a major concern in China, especially among older adults. As of 2020, China's population over the age of 60 accounted for as much as 13.5% of the total population and continues to rise (5). Older adults suffer from loneliness, diseases, disabilities, and other problems that require measures to protect their physical and mental health. Government intervention is considered an important part of the solution when people face challenges in China (5, 6). Therefore, the Chinese Communist Party Central Committee, State Council issued and implemented the “Healthy China 2030 Plan Outline” in 2016, which proposed the construction of “Healthy China,” strengthening the top-level design of health services for older adults, carrying out robust services to enhance their sense of health acquisition by older adults, and increasing the protection of health services for this population (7). To effectively manage the aging process, China implemented a pilot social pooling pension insurance program in the 1980s, deepening reforms in the 1990s. It established a fundraising model shared by the state, enterprise, and individual and unified the basic pension insurance system for enterprise employees. China's basic old-age insurance coverage is expected to reach 95% of the population by 2025 (8). China has built a comprehensive and diversified pension service system and has achieved its initial goal of providing care for older adults. With the rapid socio-economic development, the continuous increase in people's demand for a better life also promotes the continuous progress and development of pension insurance.

With the continuous increase in aging, further improvement of pension insurance is crucial to the protection of the basic life of older adults and the improvement of their physical and mental health. The redistribution effect of pension insurance on social equity narrows the gap between the rich and poor to a certain extent and alleviates the mental health problems of older adults. In addition to the economic factors such as income (9), the effect of social capital on the mental health of older adults cannot be ignored (10). Cognitive social capital refers to trust, reciprocal norms, perceived support, and social networks when providing support or resources (11, 12). Mutual trust and the formation of social networks broaden the social networks of older adults and reduce their loneliness. With sufficient trust, older adults are willing to open their hearts and improve their mental health.

In China, Confucian thought that “filial piety is the most important characteristic” that emphasizes the importance of paying attention to the physical and mental health of older adults, and pension insurance and social capital are crucial to the mental health of this population. Pension insurance can be divided into commercial and social insurance. Social capital includes cognitive and structural factors. Cognitive social capital is indispensable for daily life and closely related to the physical and mental health of older adults.

The contributions of this study are as follows: (1) It examines the impact of pension insurance and social capital on mental health, an urgent problem that needs to be solved. Analyzing these problems can provide a direction for further improving China's social security system, improving the mental health of older adults, and providing solutions to the problem of aging in other countries. (2) Previous studies have mainly explained the relationship between pension insurance and mental health or the relationship between social capital and mental health in all age groups. We specifically focus on the older adult group. The results of this study can effectively supplement and extend the existing literature on pension insurance, mental health, and social capital. (3) Through 3 years of micro-panel data to explore the influencing factors of the mental health of older adults, the pension insurance is divided into social pension insurance and commercial pension insurance, from the social and commercial, according to the difference, to explore its actual influence, make up for the previous single pension insurance.

## Literature review

### Pension insurance and the mental health of older adults

With the rapid socio-economic development and the accumulation of material wealth among residents, mental health problems, as hidden and harmful issues, have attracted worldwide attention. Many studies have measured the relationship between income, wealth, consumption, and mental health from an economic perspective (13, 14). From a macroeconomic perspective, temporary business cycle fluctuations and labor market shocks affect mental and physical health (15). In business cycle fluctuations, short-term competition can affect the labor market, while job insecurity, temporary employment, and job-related stress follow (16, 17), and the environment also affects mental health. Residents born in Denmark between 1980 and 1984 were exposed to nitrogen oxides and nitrogen dioxide for a long-time during childhood, resulting in a high risk of schizophrenia in adulthood (18). In China, Chen (19) found that PM 2.5 and PM 10 concentrations were significantly positively correlated with autism spectrum disorders.

In addition to income (or consumption and wealth) and other economic and environmental factors affecting mental health, the redistribution mechanism of pension insurance in regulating social equity has gained increasing attention. The pension insurance system, as the basic safeguard measure for residents' pension insurance, is a people's livelihood project that is combined with economic welfare socialization. Pension insurance can be divided into social and commercial types. There are differing opinions on the impact of mental health on academic sessions. Social insurance was significantly positively correlated with happiness, and residents without pension insurance had lower levels of happiness (20, 21). However, one scholar holds a negative attitude toward the mental health effects of the pension system, which is insufficient to meet the needs of life and the satisfaction of pension insurance policy and has a significant impact on rural residents' sense of happiness and social justice (22).

Commercial pension insurance, as the third pillar of personal pension, has become an important breakthrough in solving the pension problem in China. However, insufficient pension asset management, imperfect policy systems, and insufficient service demand orientation of pension financial products can be found in this system. Older people have lower insurance needs than young people (23). Simultaneously, factors such as individual age, education level (24), occupation (25), urban and rural structures (26) also affect their purchase of commercial insurance.

### Social capital

The concept of social capital was first proposed by Hanifan (27) to explain the impact of social capital on the level of school education in rural areas. Since then, social capital has been used widely in various fields. However, owing to different research perspectives, the identification of social capital varies. There are two main academic research perspectives on social capital. The first category includes both individual and collective social capital. The second category comprises of cognitive and structural social capital. The first classification distinguishes the subjects of social capital. From individual and collective levels, Putnam defines social capital as “social organization characteristics that promote mutual coordination and cooperation, such as network and social trust,” and regards it as a community-level resource (28). Under this classification, social capital is regarded as collective characteristic research, which usually measures the level of social capital by aggregating the cohesion perception or social interaction mode between members (29) in a macro-environment.

Individual social capital focuses on social networks at the individual level. Bourdieu defined social capital as “the sum of actual or potential resources related to a lasting network with institutionalized mutual understanding and recognition” and focuses on the resources accumulated at the individual level as a result of joining the social network (30). He considers social capital to be an individual asset that can be tested through individual social network relationships and that individuals can have high or low levels of social capital at the individual level. This depends on the network status or the degree of trust and reciprocity that individuals perceive (31).

The second classification no longer distinguishes the subject of social capital but looks at the field from the content of social capital divided into cognitive and structural social capital (32, 33), the structural component of social capital within social structures such as networks and associations, focusing on behavior (such as that within participating associations), and members of social organizations (31). The cognitive component of social capital is related to its subjective and intangible characteristics, such as trust and reciprocal norms in the provision of support or resources, perceived support, and social networks (11, 12).

## Theoretical analysis and assumptions

Social pension insurance is a public welfare insurance jointly funded by society, enterprises, and individuals. The basic old-age security provided to older adults by social pension insurance is referred to as a pension. The receipt of a pension greatly alleviates people's economic and lifestyle burdens and promotes the mental health of older adults. When older people leave the labor market, the loss of labor income becomes entangled with various diseases and has an inevitable impact on individuals' physical and mental health. The social insurance system reduces the incidence of poverty among older adults by moderately adjusting the income levels of different classes, thereby indirectly improving their health (34). This indicates a certain degree of social redistribution. The public welfare and sociality of social pension insurance are conducive to improving the subjective wellbeing of older adults and promoting their mental health (35). Hence, Hypothesis 1 is as follows.

H1: There is a significant positive correlation between social pension insurance and mental health of older adults.

As a supplement to social pension insurance, commercial pension insurance is a type of for-profit insurance in which residents voluntarily participate at their own expense. Commercial pension insurance is divided into four categories: traditional pension, dividend pension, universal pension, and investment connections. Traditional pension insurance is a type of saving insurance in which individuals gain benefits through interest rates and have a low level of risk. However, in periods of inflation, the depreciation of the inflation rate is likely to be relatively high. Dividend-based pension insurance can avoid inflation risks to a certain extent; however, the annual dividends of a company are based on the operations of the insurance company, which is unpredictable. Universal pension insurance requires flexible access and financial vulnerability and has a significant impact on residents' insurance purchases (23). It is difficult for people with low levels of self-control to experience pension effects. Investment pension insurance is the riskiest type of commercial pension insurance, and it cannot withstand short-term fluctuations and is not suitable for pension insurance. Therefore, commercial pension insurance is an important financial product. When older adults are insured, they are inevitably affected by uncertainty and risk, which are not conducive to their mental health. Thus, Hypothesis 2 is as follows.

H2: There is an obvious inverse relationship between commercial pension insurance and mental health of older adults.

The cognitive components of social capital are related to its subjective and intangible aspects, such as trust and reciprocal norms, perceived support, and social networks when providing support or resources (11, 12). This is related to people's feelings. Research on aging shows that social capital keeps older adults in a society as healthy, independent, and positive (35, 36). Social capital allows individuals to fully trust others, provide sense support, and form social networks. Older adults, when their children leave their nest and society continues to develop, inevitably feel lonely and disconnected from society. When older adults have good social capital, they are more willing to leave their homes to socialize. Although pension insurance can affect the mental health of this population to a certain extent, it weakens its impact of pension insurance on mental health. Therefore, we propose the following hypotheses:

H3-1: Social capital significantly weakens the impact of social pension insurance on mental health.
H3-2: Social capital significantly weakens the impact of commercial pension insurance on mental health.

## Research design and analysis

### Data source and research design

The data used in this study were from China Family Panel Studies. Institutions such as the Peking University of China and the Center for Investigation and Research at the University of Michigan in the United States cooperate to collect, collate, and publish these data. The CFPS is a national and comprehensive social tracking survey project that aims to reflect the changes in society, economy, population, education, and health in China by tracking and collecting data from three levels: individuals, families, and communities, and provides a data foundation for academic and policy research. These data were obtained from a 2-year follow-up survey. Based on the purpose of this study, adult questionnaires used in 2016, 2018, and 2020 were selected for inclusion. According to the World Health Organization and the “Law of the People's Republic of China on the Protection of the Rights and Interests of Older Adults,” people over 60 years old are considered as “older adults.” Therefore, this study selected data from individuals over 60 years of age. After eliminating invalid data (missing, invalid, or refusal to answer), 8,979 valid samples were obtained.

### Variable description

#### Dependent variable: Mental health of older adults

The Center for Epidemiological Study Depression (hereinafter referred to as CESD) scale was used in the adult database of the CFPS. It was used to assess the mental health of older adult participants. A tracking survey in China compressed the CEDS into eight problems in 2018 and 2020. The study uses eight indicators of CESD: “I live happily” and “I feel happy.” The answers to these two questions are “almost never (less than 1 day per week),” “sometimes (1–2 days per week),” “often (3–4 days per week),” and “most of the time (5–7 days per week),” which are assigned zero, one, two, and three points, respectively. The answers to the six questions, that is, “I feel depressed,” “I have difficulty doing anything,” “My sleep is not good,” “I feel lonely,” “I feel sad,” and “I feel life can't go on,” were “almost never (less than 1 day per week),” “sometimes (1–2 days per week),” “often (3–4 days per week),” and “most of the time (5–7 days per week).” They were assigned three, two, one, and zero points, respectively. Finally, the scores of the eight questions were added to obtain the participants' mental health indices (37).

#### Independent variables: Social pension and commercial pension insurance

“Social pension insurance” is defined as a social insurance project jointly funded by the state and individuals and includes “retirement pension (mainly from financial allocation),” “basic pension insurance,” “enterprise supplementary pension insurance,” “new rural social pension insurance,” and “urban residents' pension insurance.” In the CFPS, the existence of this insurance is assigned one while “none of the above” is assigned zero. Commercial pension insurance is mainly personally funded and includes “commercial pension insurance” and “rural pension insurance.” In CFPS, the existence of this insurance is assigned one while “none of the above” is assigned zero.

#### Regulatory variable: Social capital

Social capital is generally divided into structural and cognitive (32). Cognitive capital is mainly related to personal perceptions such as trust and reciprocity when providing support or resources (14, 31). This study primarily discusses the influence of social capital on the mental health of older adults. Based on the cognitive social capital structure, two indicators were processed in the CFPS. The first asked whether the participants were willing to help or selfish. Most people in A were willing to help others, and most people in B were selfish. The values of those who chose answers A and B were one and zero, respectively. Trust was measured as follows: generally speaking, do you think you can trust most people, or do you think it is better to be more careful with people? Most of the people in A are trustworthy. The value of the population choosing answer A was assigned one, and the value of answer B was assigned zero. Two indicators were added, and cognitive social capital was obtained.

#### Control variables

Personal characteristics, including age, income status, marriage, exercise, and so on. The definitions and expressions of the variables are listed in Table 1.

**Table 1:** Variable definition.

**Variable**	**Variable name**	**Symbol**	**Definition**
Dependent variable	Mental health of older adults	Mental	“I live happily,” “I feel happy,” The answers to these two questions are “almost never (less than 1 day per week),” “sometimes (1–2 days per week),” “often (3–4 days per week),” “most of the time (5–7 days per week),” which are assigned 0, 1, 2, and 3 points respectively. “I feel depressed,” “I have difficulty doing anything,” “My sleep is not good,” “I feel lonely,” “I feel sad,” and “I feel life can't go on.” The answers to these six questions are “almost never (less than 1 day per week),” “sometimes (1–2 days per week),” “often (3–4 days per week),” “most of the time (5–7 days per week),” which are assigned 3, 2, 1 and 0 points respectively. Finally, the scores of the eight questions were added to obtain the participants' mental health index
Independent variable
Pension insurance	Social pension insurance	Social	In this study, the social pension insurance is defined as a social insurance project jointly funded by the state and individuals, including “retirement pension (mainly from the financial allocation),” “basic pension insurance,” “enterprise supplementary pension insurance,” “new rural social pension insurance,” “urban residents pension insurance.” Seniors who participate in the above insurance are assigned a value of 1; those who do not participate in the above insurance are assigned a value of 0
	Commercial pension insurance	Commercial	Commercial pension insurance is mainly personally funded insurance, including “commercial pension insurance” and “rural pension insurance.” Seniors who participate in the above insurance are assigned a value of 1; those who do not participate in the above insurance are assigned a value of 0
Regulated variable	Cognitive social capital	Capital	The first is: do you think most people are helpful or selfish? The answer is that most people are willing to help, scoring 1. The answer is that most people are selfish, scoring 0; the second question is, in general, do you think most people are trustworthy or more careful with others? Answer Most people are trustworthy, scored 1, the more careful the better, scored 0; finally, the scores of these two problems are summed to obtain cognitive social capital
Control variable
	Age	Age	Age of respondents
	Gender	Gender	Man = 1, Woman = 0
	Income status	Income	“Your personal income belongs locally to _?” The scoring range is 1–5, from low to high. In this article, the groups divided into 1–2 points are divided into low-income groups, assigned to 0, and the groups divided into 3–5 are divided into high-income groups, assigned to 1
	Marital status	Marital	Responses to “Current marital status?” The answers are: unmarried, spouse (married), cohabiting, divorced, widowed. Spouse (married) are classified as married and assigned a value of 1, while unmarried, cohabiting, widowed and divorced are classified as unmarried and assigned a value of 0
	Exercise	Exercise	In the past 12 months, under normal circumstances, how many minutes per exercise?
	Health status	Health	the question “What is the current state of health?,” healthy = 0 and unhealthy = 1
	Smoking	Smoking	Did you smoke a month ago? Yes is 1, No is 0
	Drinking	Drinking	Do you drink more than three times a week in the past month? Yes is 1, No is 0

### Model construction

To empirically examine the relationship between pension insurance and the mental health of older adults, as well as the moderating effect of social capital, this study selected panel data from 2016, 2018, and 2020. Missing variables such as the unobservable heterogeneity of individuals, families, and regions may affect the empirical results and lead to estimation errors. Therefore, this study mainly used the random effect estimation method (random effect, RE) and the feasible generalized least squares estimation method (FGLS) to control for the heterogeneity. Simultaneously, the results of the ordinary least square estimation method (OLS) under the mixed panel are reported for comparison (37) and as one of the robustness tests. This study considers the mental health of older adults as dependent variables and social and commercial pension insurance as independent variables and controls for the relevant personal characteristics to set up Model (1):


(1) $$\begin{array}{l} Mental_{it}=\alpha_{1}{\text{Social}}_{it}+\alpha_{2}{\text{Commercial}}_{it}\\ \;+\alpha_{i}X_{it}+u_{i}+\varepsilon_{it} \end{array}$$

To test the moderating effect of social capital on pension insurance and mental health of older adults, the following model was established:


(2) $$\begin{array}{l} Mental_{it}=\alpha_{1}{\text{Social}}_{it}+\alpha_{2}{\text{Commercial}}_{it}+\alpha_{3}{\text{Social}}_{it}\;*\;\\ {\text{ Capital}}_{it}+\alpha_{i}X_{it}+u_{i}+\varepsilon_{it} \end{array}$$

(3) $$\begin{array}{l} Mental_{it}=\alpha_{1}\text{Social}+\alpha_{2}{\text{Commercial}}_{it}++\alpha_{4}\;\\ {\text{ Commercial}}_{it}l\;*\;{\text{Capital}}_{it}+\alpha_{i}X_{it}+u_{i}+\varepsilon_{it} \end{array}$$

*Mental*_*it*_ was the explained variable, indicating that participants were mentally healthy. The greater the value of *Mental*_*it*_, the healthier older adults are mentally. i represents an individual; t represents 2016, 2018, or 2020; *Social*_*it*_ and *Commercial*_*it*_ are the explanatory variables, indicating the status of social and commercial pension insurance; *Capita*_*it*_is the moderator variable, indicating social capital. *X*_*it*_ includes a set of control variables that change with individuals and time, such as age, income status, and marriage, and those that change with individuals but do not change with time, such as gender. The disturbance term was composed of (*u*_*i*_+ε_*it*_), where the unobservable random variable *u*_*i*_ is the individual heterogeneity, and ε_*it*_ is the error term variable that changes with the individual and time. Based on the AIC test, the minimum AIC value of Model 2 was 49,699.55, and Model 2 was the best among the three models. However, to test for the impact of social and commercial pension insurance on the mental health of older adults as well as the moderating effect of social capital, the regression results of the three models are still considered in this study.

## Empirical analysis

### Descriptive analysis

The descriptive statistics of the ratio scale variables are shown in terms of the minimum and maximum values, mean, and standard deviation. The categorical variables were described as counts and percentages. As shown in Table 2, the maximum value for mental health was 24, the minimum value was 0, and the average value was 18.1, which shows that the mental health of older adults in China has a positive trend. The proportion of social pension insurance is 46.3%, and the number of people who do not participate in insurance has been reduced. Thus, the data were more balanced. However, the number of people participating in commercial pension insurance was lower at 3.3, indicating that the penetration rate of commercial pension insurance for adults over 60 years of age in China was not high. Compared to the commercial pension insurance, more people participate in social pension insurance. In the case of joint investment by society and individuals, people's economic burden was greatly reduced, and they are more willing to invest in social pension insurance. The value for cognitive social capital ranges from 0 to 2, with a mean of 1.29. This figure indicates that people generally trust others and are willing to help others. This study examined the mental health of older adults. The age of the sample was over 60 years, and the average value was 67.6, showing that there were more people who had just begun their older adult life. In this age conversion, it is more valuable to examine the changing trends in mental health. The proportion of men was 50.5%, and the proportion of women was 49.5%. Altogether, 80.9% were married and 19.1% were unmarried.

**Table 2:** Descriptive statistics.

**Variable**	**Proportion**	**Mean**	**Std. Dev**.	**Min**	**Max**	**Obs**
**Continuous variables**
Mental		18.19	4.043	0	24	8,979
Cognitive social capital		1.29	0.777	0	2	8,979
Age		67.63	6.504	60	98	8,979
Exercise		3.47	3.66	0	35	8,979
Categorical variables						
Social pension insurance						8,979
1	46.3					4,161
0	53.7					4,818
Commercial pension insurance						8,979
1	3.3					8,684
0	96.7					295
Gender						8,979
1	50.5					4,537
0	49.5					4,442
Income						8,979
1	55.7					5,005
0	44.3					3,974
Marital						8,979
1	80.9					7,263
0	19.1					1,716
Health						8,979
1	50.3					4,513
0	49.7					4,466
Smoking						8,979
1	29.1					2,615
0	70.9					6,364
Drinking						8,979
1	17.0					1,522
0	83.0					7,457

Drinking and smoking were reported by 17 and 29% of the sample, and the older adults had healthy living conditions. The average exercise time was 3.47 min. The definition of exercise is the time of each exercise under normal conditions in the past 12 months. Because the data were selected from adults over 60 years old and due to the limitations of physical condition, older adults had a shorter time to exercise each time, especially the indoor and outdoor physical activities, mainly for the purpose of physical fitness and pleasure. The “Healthy China” policy was implemented in 2016, and the awareness of older adults to exercise was low, so the average value was low.

### Regression analysis

#### Impact of pension insurance on the mental health of older adults

In Table 3, Model 1 estimates the impact of pension insurance on the mental health of older adults in the total sample. Column 1 shows the OLS regression result. Column 2 reports the panel random effect estimation method and the feasible generalized least squares estimation method (Panel + FGLS) regression results. The comparison shows that the results of the two estimation methods were relatively close. The regression results of Panel + FGLS are mainly reported and analyzed below. The coefficient of social pension insurance was 0.267. The coefficient which is greater than 0 has a significant positive correlation with the mental health of older adults. When people participate in social pension insurance, their psychology is healthier, and the mental health of the older adults is promoted at the level of 1%, which is similar the conclusion of Galiani and Gertler (38). Social pension insurance is jointly funded by the society and individuals, which has certain public welfare and social elements. With the support of society, the daily life of the older adults after purchasing the social pension insurance is guaranteed. In China, men over 60 years of age and women over 55 years of age can apply for pensions. The receipt of pensions greatly reduces economic pressures. The older adults improve their mental health by increasing their sense of psychological acquisition and income distribution effects (34).

**Table 3:** Regression results of pension insurance on mental health of older adults.

**Variable**	**Model 1**			
	**OLS**		**Panel**+**FGLS**	
Social pension insurance	0.306***	0.276***	0.277***	0.267***
	(0.086)	(0.082)	(0.084)	(0.080)
Commercial pension insurance	−0.485***	−0.775***	−0.522**	−0.764***
	(0.242)	(0.230)	(0.247)	(0.223)
Age		0.008		0.007
		(0.006)		(0,006)
Gender		0.771***		0.782***
		(0.098)		(0.101)
Income		0.512***		0.486***
		(0.083)		(0.082)
Marital		1.235***		1.267***
		(0.109)		(0.113)
Exercise		0.056***		0.047***
		(0.011)		(0.011)
Health		1.826***		1.760***
		(0.082)		(0.082)
Smoking		−0.374***		−0.349***
		(0.104)		(0.107)
Drinking		0.476***		0.442***
		(0.115)		(0.117)
Obs	8,979	8,979	8,979	8,979
R2 /R2 between	0.0021	0.1036	0.0018	0.1198

*, **, and ***: significance at the 10%, 5%, and 1% levels, respectively.

( ) Standard error.

The coefficient of commercial pension insurance was −0.764, showing that it was negatively correlated with the mental health of the older adults and was significant at the 1% level. Commercial insurance differs from social insurance in that it is profitable and risky. China's commercial pension insurance coverage is low and has little impact on the mental health of older adults. Commercial pension insurance is associated with defects and risks. Although commercial pension insurance has a certain income, the existence of risk leads anxiety among older adults. Owing to the high price of commercial pension insurance products and relatively low level of protection, the current value of pensions obtained by this population is less than half of the current value of their contributions, which is not conducive to the development of mental health in older adults.

From the panel + FGLS regression, being male, income status, marital status, exercise time, health status, and drinking were positively correlated with mental health and were significant at the 1% level. The higher the income of older adults, the better the living standard, the lower the economic pressure, the stronger the happiness, and the better the mental health (39). The married state of older adults, good exercise status, and positive evaluation of self-health are conducive to the development of mental health in older adults. Exercise strengthens physical health and thus improves mental health. The loneliness of unmarried status and fear of negative self-evaluation of health are not conducive to the development of mental health in older adults. smoking was significantly negatively correlated at the 1% level. Smoking is harmful to health, especially in older adults with poor immunity, and is not conducive to lung health.

#### Regulatory effects of social capital

Models 2 and 3 in Table 4 estimate the moderating effect of social capital on social pension insurance and commercial pension insurance for the total sample and report the regression results of the panel and FGLS. In the regression results of Model 2, after introducing the interaction term between social capital and social pension insurance, the regression coefficient of the interaction term was 0.418 and was significant at the level of 1%, indicating that it has a significant regulatory effect. Social capital enhances the promoting effect of social pension insurance on the health of older adults. When social capital is added, older adults have strong social self-confidence and mutual assistance. Under cognition, older adults are willing to leave the house, communicate with others, conduct social and consumer activities, and improve their mental health. Social pension insurance is the basic social security for older adults and is mainly used to improve the basic income. When income and interpersonal relationships improve, so does mental health.

**Table 4:** Regression results of moderating variables.

**Variable**	**Model 2**		**Model 3**	
	**OLS**	**Panel + FGLS**	**OLS**	**Panel+FGLS**
Social pension insurance	−0.272**	−0.272**	0.274***	0.267***
	(0.128)	(0.126)	(0.082)	(0.080)
Commercial pension insurance	−0.780***	−0.770***	−1.456***	−1.335***
	(0.229)	(0.223)	(0.402)	(0.392)
Social*Capital	0.423***	0.418***		
	(0.076)	(0.075)		
Commercial*Capital			0.570**	0.476*
			(0.276)	(0.269)
Age	0.006	0.005	0.008	0.006
	(0.006)	(0.006)	(0.006)	(0.006)
Gender	0.770***	0.780***	0.772***	0.783***
	(0.097)	(0.101)	(0.098)	(0.101)
Income	0.502***	0.478***	0.511***	0.485***
	(0.083)	(0.082)	(0.083)	(0.082)
Marital	1.237***	1.270***	1.233***	1.264***
	(0.109)	(0.113)	(0.109)	(0.113)
Exercise	0.056***	0.047***	0.056***	0.047***
	(0.011)	(0.011)	(0.011)	(0.011)
Health	1.805***	1.739***	1.823***	1.758***
	(0.082)	(0.082)	(0.082)	(0.082)
Smoking	−0.375***	−0.349***	−0.374***	−0.349***
	(0.104)	(−0.107)	(0.104)	(0.107)
Drinking	0.481***	0.449***	0.480***	0.445***
	(0.115)	(0.117)	(0.115)	(0.117)
Obs	8,979	8,979	8,979	8,979
R2 /R2 between	0.1065	0.1231	0.1039	0.1200

*, **, and *** significance at the 10, 5, and 1% levels, respectively.

( ) Standard error.

In the regression results of Model 3, after introducing the interaction term between social capital and commercial pension insurance, the regression coefficient of the interaction term was 0.476, which was significant at the level of 10%, indicating that it has a significant moderating effect and alleviates the negative shadow effect of commercial pension insurance. Social capital, such as mutual trust, regional security, and high social reciprocity, can reduce the incidence of depression and improve mental health, thereby alleviating the negative impact of commercial pension insurance (40).

### Follow-up testing

#### Model substitution method

To test for the internal mechanism of mental health among older adults in social and commercial pension insurance and the moderating effect of social capital, this study uses mixed OLS regression. The specific results are shown in Tables 3, 4. The test results are consistent with the panel and FGLS regression results and pass the robustness test.

#### Short time window

In 2020, the new coronavirus epidemic swept the world, resulting in the slow development of the entire socio-economic, cultural, sports, health, and other aspects. Owing to the poor physical immunity of older adults, there are problems such as reduced social activities, exercise time, and income. Therefore, to eliminate the data interference caused by the epidemic, the data were adjusted to 2016 and 2018, and panel and FGLS regression was re-performed. The regression results are presented in Table 5. After the test, it was found that social pension insurance was proportional to the mental health of older adults, and commercial pension insurance was inversely proportional to the mental health of older adults, which is consistent with the previous regression, but the significance changed from 1 to 5 %. The adjustment effect of social capital on social pension insurance and older adults did not change, but the adjustment effect of social capital on commercial pension insurance and mental health of older adults was not significant.

**Table 5:** Regression results for narrowing time window.

**Variable**	**Model 1**	**Model 2**	**Model 3**
Social pension insurance	0.213**	−0.309**	0.213**
	(0.084)	(0.131)	(0.084)
Commercial pension insurance	−0.567**	−0.578**	−1.054**
	(0.280)	(0.280)	(0.471)
Social*Capital		0.404***	
		(0.078)	
Commercial*Capital			0.436
			(0.339)
Age	0.007	0.005	0.007
	(0.006)	(0.006)	(0.006)
Gender	0.763***	0.763***	0.764***
	(0.105)	(0.105)	(0.105)
Income	0.533***	0.525***	0.533***
	(0.086)	(0.086)	(0.086)
Marital	1.267***	1.270***	1.266***
	(0.117)	(0.117)	(0.117)
Exercise	0.080***	0.080***	0.080***
	(0.011)	(0.011)	(0.011)
Health	1.770***	1.751***	1.768***
	(0.086)	(0.086)	(0.086)
Smoking	−0.313***	−0.316***	−0.314***
	(0.112)	(0.111)	(0.112)
Drinking	0.440***	0.448***	0.440***
	(0.123)	(0.122)	(0.123)
Obs	7,978	7,978	7,978
R2 /R2 between	0.1217	0.1248	0.1220

*, **, and *** significance at the 10, 5, and 1% levels, respectively.

( ) Standard error.

#### Missing variables

To make the research results of this study reliable, urban and rural variables were added to the stability test. The differences between urban and rural areas, living environment, economic environment, infrastructure, and other aspects may affect the mental health of older adults in all aspects. Older adults living in urban areas were set to one, and those living in rural areas were set to zero. The panel and FGLS regressions were re-conducted. The test results are presented in Table 6.

**Table 6:** Regression results of adding missing variables.

**Variable**	**Model 1**	**Model 2**	**Model 3**
Social pension insurance	0.255***	−0.297**	0.255***
	(0.089)	(0.126)	(0.080)
Commercial pension insurance	−0.686***	−0.692***	−1.269***
	(0.221)	(0.221)	(0.388)
Social*Capital		0.427***	
		(0.075)	
Commercial*Capital			0.488*
			(0.267)
Age	0.004	0.002	0.004
	(0.006)	(0.006)	(0.006)
Gender	0.760***	0.758***	0.761***
	(0.100)	(0.100)	(0.100)
Income	0.484***	0.475***	0.483***
	(0.081)	(0.081)	(0.081)
Marital	1.272***	1.277***	1.269***
	(0.112)	(0.112)	(0.112)
Exercise	0.027**	0.027**	0.027**
	(0.011)	(0.011)	(0.011)
Health	1.753***	1.732***	1.750***
	(0.082)	(0.082)	(0.082)
Smoking	−0.283***	−0.282***	−0.283***
	(0.106)	(0.106)	(0.106)
Drinking	0.396***	0.403***	0.399***
	(0.116)	(0.116)	(0.116)
Rural-urban	1.086***	1.090***	1.087***
	(0.084)	(0.084)	(0.084)
Obs	8,946	8,946	8,946
R2 /R2 between	0.1377	0.1413	0.1380

*, **, and ***: significance at the 10, 5, and 1% levels, respectively.

( ) Standard error.

## Conclusion

### Results

The differentiation in pension insurance has different effects on the mental health of older adults. Social pension insurance is proportional to the mental health of older adults. When older adults participate in social pension insurance, the mental health of older adults is better. Commercial pension insurance is inversely proportional to the mental health of older adults. When older adults participate in commercial pension insurance, it is not conducive to the mental health of older adults. Social capital has a significant regulatory effect on commercial and social pension insurance. It enhances the promoting effect of social pension insurance on the health of older adults. Social capital alleviates the negative shadow effect of commercial pension insurance.

### Discussion

#### Pension insurance and mental health of older adults

Social pension insurance alleviates the anxiety and negative emotions of older adults and improves their mental health. To a certain extent, public welfare and the pensions alleviate the living pressure of older adults and the sense of social equity in income redistribution and greatly promotes their mental health of older adults. However, commercial pension insurance is not conducive to improving the mental health of older adults, and this finding differs from previous research results. Previous research has mainly focused on the beneficial effects of commercial pension insurance on the mental health of older adults or analyzes the relationship between commercial pension insurance and other variables. The main reasons are as follows: First, the penetration rate of China's commercial pension insurance is low, and most older adults in China choose social pension insurance as the basic guarantee for older adult life; and second, commercial pension insurance is risky. Older adults who purchase commercial pension insurance have concerns, anxiety, and so on, as well as a lack of understanding and information channels for commercial pension insurance, which has a negative impact on commercial pension insurance and is not conducive to the development of mental health in older adults.

#### Social capital

This study identifies social capital as cognitive social capital, which is divided into trust in residents and mutual assistance. Social capital enhances the promoting effect of social pension insurance on the health of older adults, weakening the adverse effects of commercial pension insurance. Older adults with high levels of trust and mutual assistance were more willing to leave their homes for social interaction. One type of social interaction involves obtaining explicit and invisible commercial pension insurance information from the residents. Residents need to make cost, risk, and benefit judgments in the process of purchasing commercial pension insurance and often face insufficient information. Access to social interaction information effectively meets residents' needs, thereby reducing the risk of commercial pension insurance. Second, the development of social interactions and community activities increased the daily activities of older adults. During interpersonal communication, the body and mind are relaxed. With the relief of loneliness and a sense of entertainment, mental health is effectively improved and the adverse effects of commercial pension insurance are alleviated. It also enhances the positive effect of social pension insurance on the mental health of older adults.

### Suggestions

#### Pension insurance and the mental health of older adults

Under the active role of social pension insurance, China should further popularize social pension insurance, take social pension insurance as a basic step toward social security, benefit the people, improve the health of the entire population, and lay a solid foundation for the implementation of the Healthy China strategy. As a supplement to social pension insurance, commercial pension insurance is a type of profit-making insurance in which residents voluntarily participate at their own expense. However, in China, the replacement rate of commercial pension insurance, which is the third pillar of social pension insurance, is low. The risk of commercial pension insurance is still a point at which buyers focus excessively. Lack of understanding and distrust of commercial pension insurance are not conducive to its development. Therefore, commercial pension insurance should be promoted to residents through the mass media and other channels.

#### Social capital

China needs to strengthen community building and the development of the silver economy industry, promote infrastructure construction for older adults, and provide convenient places for older adults to travel to and to which they can be consumers. Through mass media, older adults can promote the country's policies for older adults and improve their social trust, social interactions, and mental health.

### Limitations and recommendations for future research

This research represents only a beginning rather than an end. This study only preliminarily establishes the correlation between commercial pension insurance, social pension insurance, social capital, and mental health of older adults, and a more detailed analysis of the mechanism of action needs to be further explored. The data selection is limited to only cognitive social capital in this study, and the correlation between collective social capital and other personal social capital and the health status of older adults needs to be verified again. In addition, there are other factors that affect the mental health of older adults, such as the living environment, which need to be analyzed in depth. Therefore, in future, more rigorous and detailed research should be conducted to further explore the mental health status of older adults and improve and optimize policies based on existing results.

## Data availability statement

The original contributions presented in the study are included in the article/Supplementary material, further inquiries can be directed to the corresponding author.

## Ethics statement

Ethical review and approval was not required for the study on human participants in accordance with the local legislation and institutional requirements. Written informed consent from the patients/participants or patients/participants legal guardian/next of kin was not required to participate in this study in accordance with the national legislation and the institutional requirements.

## Author contributions

K-CC contributed to the conception of the study and directed essay writing. QL gathered information and contributed significantly to analysis and manuscript preparation. CJ and Y-JL performed the data analyses and wrote the manuscript. ZC collected and analyzed data. XH helped perform the analysis with constructive discussions. All authors contributed to the article and approved the submitted version.

## Funding

This study was funded by the National Social Science Foundation of China (21BZZ026).

## Conflict of interest

The authors declare that the research was conducted in the absence of any commercial or financial relationships that could be construed as a potential conflict of interest.

## Publisher's note

All claims expressed in this article are solely those of the authors and do not necessarily represent those of their affiliated organizations, or those of the publisher, the editors and the reviewers. Any product that may be evaluated in this article, or claim that may be made by its manufacturer, is not guaranteed or endorsed by the publisher.
